# Neonatal high protein intake enhances neonatal growth without significant adverse renal effects in spontaneous IUGR piglets

**DOI:** 10.14814/phy2.13296

**Published:** 2017-05-29

**Authors:** Farid Boubred, Agnes Jamin, Christophe Buffat, Laurent Daniel, Patrick Borel, Gaëlle Boudry, Isabelle Le Huëron‐Luron, Umberto Simeoni

**Affiliations:** ^1^ NORT Aix‐Marseille Université INRA INSERM Marseille France; ^2^ INRA UR1341 ADNC Saint‐Gilles France; ^3^ Laboratoire de biologie moléculaire AP‐HM Marseille France; ^4^ UPRES EA3281 Faculté de Médecine Aix‐Marseille Université Marseille France; ^5^ DOHaD Laboratory CHUV University Hospital and UNIL Lausanne Switzerland

**Keywords:** Catch‐up growth, glomerular sclerosis, high protein intake, intrauterine growth restriction, low birth weight, nephron number

## Abstract

In humans, early high protein (HP) intake has been recommended to prevent postnatal growth restriction and complications of intrauterine growth restriction (IUGR). However, the impact of such a strategy on the kidneys remains unknown, while significant renal hypertrophy, proteinuria, and glomerular sclerosis have been demonstrated in few experimental studies. The objective of this study was to evaluate the effects of a neonatal HP formula on renal structure in IUGR piglets. Spontaneous IUGR piglets were randomly allocated to normal protein (NP,* n* = 10) formula or to HP formula (+50% protein content, *n* = 10) up to day 28 after birth. Body weight, body composition, renal functions, and structure were assessed at the end of the neonatal period. While birth weights were similar, 28‐day‐old HP piglets were 18% heavier than NP piglets (*P *<* *0.01). Carcass protein content was 22% higher in HP than in NP offspring (*P *<* *0.01). Despite a HP intake, kidney weight and glomerular fibrosis were unaltered in HP piglets. Only a 20% increase in glomerular volume was noted in HP piglets (*P* < 0.05) and restricted to the inner cortical area nephrons (*P *=* *0.03). Plasma urea/creatinine ratio and proteinuria were unchanged in HP piglets. In conclusion, neonatal HP feeding in IUGR piglets significantly enhanced neonatal growth and tissue protein deposition but mildly affected glomerular volume. It can be speculated that a sustained tissue protein anabolism in response to HP intake have limited single nephron glomerular hyperfiltration.

## Introduction

Low birth weight (LBW) infants, especially those suffering from intrauterine growth restriction (IUGR), frequently displayed postnatal growth restriction during the neonatal period and early infancy (Agostoni et al. 2010). Early postnatal growth restriction affects morbidity and long‐term neurocognitive functions (Ehrenkranz et al. 2011; Sammallahti et al. 2014). Improving nutrition including early high protein (HP) intake has been proposed to prevent these adverse effects (Agostoni et al. 2010). However, the impact of such a nutritional strategy on the kidneys has been insufficiently studied especially in LBW infants as well as in IUGR animals (Boubred et al. 2009, 2016; Luyckx et al. 2009; Weintraub et al. 2015). Based on experimental studies and few human evidence, high protein intake is known to induce renal hypertrophy, proteinuria, and glomerular sclerosis through a single nephron glomerular hyperfiltration (SNGHF) (Brenner et al. 1982; King and Levey 1993; Slomowitz et al. 2004). These effects are amplified when the nephron number is reduced as in IUGR offspring (Brenner et al. 1982; Boubred et al. 2009). Renal filtered amino‐acid overload inhibits tubule‐glomerular feedback responsible for glomerular vasodilation and SNGHF (Brenner et al. 1982; King and Levey 1993). In IUGR rat offspring, we and others have shown that early neonatal overfeeding or an elective neonatal high protein diet affect renal structure and functions as early as during the neonatal period with significant renal hypertrophy, renal overweight, proteinuria, and glomerular sclerosis (Bassan et al. 2000; Boubred et al. 2009, Luycks et al. 2009). However, extrapolating these last findings to human remains questioned since renal physiology in the rat pups obviously differ from newborn infants. For instance in contrast with piglet and humans, nephrogenesis continues after birth up to day 7–10 in the rat and can be influenced by postnatal factors (Boubred et al. 2009). Therefore, we used in this study a large animal model with spontaneous IUGR in piglets which is a suitable model for newborn IUGR infants (Bauer et al. 2003). Also, we aimed to assess whether HP feeding of spontaneous IUGR piglets affected short‐term renal structure.

## Materials and Methods

### Animals and diet

The care and use of pigs were performed in compliance with the French Ministry of Agriculture and Fisheries’ guidelines and with the European guidelines for animal experiments (EU Directive/63/EU). Cross‐bred [Piétrain x (Large White x Landrace)] full‐term IUGR piglets were obtained from the experimental herd of INRA (Saint‐Gilles, France) and followed the experimental protocol as previously described (Sarr et al. 2011). IUGR was defined by a birth weight below the 10th percentile which was calculated from the overall herd birth weight. In this model, IUGR is caused by placenta insufficiency due, in part, to limited vascular supply in border zone region located at the crossing of second and third part of the uterine horns (Bauer et al. 2003). Briefly, at postnatal day 2, pairs of littermates (males and females) from different litters were randomly allocated to two artificially reared groups. Animals were allowed to suckle colostrum from their mothers for the first 2 days. One piglet of each pair received a normal protein formula (NP, *n* = 10) and the other one a high protein formula (HP, *n* = 10). After separation from their mothers, from day 2 to day 7, they were placed individually in incubators (33°C, 60% humidity) and were bottle‐fed every 2 h from 07 am to 11 pm and once during the night at 03 am. At day 7, piglets were transferred into individual cages equipped with an automatic device delivering milk 10 times a day (same schedule) in a temperature‐controlled room at 30°C until d 28 (the end of the suckling period), the end of the experiments.

Piglets received a milk formula to provide the same amount of nutrients as sow milk (NP) or a high protein formula (HP) (Sarr et al. 2011). The HP formula contained 7.7 g/100 mL of proteins, 7.9 g/100 mL of lipids, and 4.6 g/100 mL of carbohydrates with total energy of 120 Kcal/100 mL versus 5.1 g/100 mL, 8.2 g/100 mL, and 4.9 g/100 mL with 113 Kcal/100 mL in NP formula, respectively. Both formula powders were manufactured by the Laiterie de Montaigu (Montaigu, France). A NP formula was formulated using whey protein concentrate, skimmed milk powder, and potassium caseinate as main sources of proteins. The HP formula was designed to provide a 40% higher amount of proteins per day but the same casein/whey proteins ratio and fat to carbohydrates ratio. The increase in protein content of the HP formula was achieved by increasing the amount of whey protein concentrate and potassium caseinate. The amount of proteins offered to the HP piglets was progressively increased (30% higher than NP piglets from d2 to d7 and 40% higher than NP piglets from d8 onward) to prevent feeding intolerance. This was achieved by offering the HP piglets, a milk formula prepared by mixing NP and HP formulas (30/70 v/v) from d2 to d7. The individual daily milk quantity offered to the piglets was calculated to provide 312 kcal/kg of metabolic weight (body weight^0.75^) per day. Milk formulas were prepared daily from powders, maintained at 4°C, and then warmed up before meal. Body weights (15 min before feeding) were recorded daily from day 2 to day 7, and then twice a week from day 9 to day 28.

### Sample collection

At day 28, the animals were killed by electronarcosis followed by exsanguination. Blood and urine samples were immediately collected, transferred to heparinized tubes, then centrifuged. Once collected, plasma was stored at −20°C for later analysis. Plasma creatinine, urea, protein concentrations and urinary creatinine and protein concentrations were measured by a standard autoanalyzer (Synchron LX20 autoanalyzer, Beckman Coulte, Brea, CA). The Jaffe method was used to determine creatinine concentrations. The heart, liver, kidneys, lungs, peri‐visceral adipose tissue, digestive, and reproductive tracts were removed. Peri‐renal adipose tissue (PRAT) and semi‐tendinus muscle were collected and weighed. The right half of the carcass (without PRAT, head, and tail) was then weighed and minced. A sample was taken, freeze‐dried, and ground into a uniform fine powder for dry matter determination and further chemical analyses. All analyses were then performed in duplicates. The dry matter content was measured after freeze‐drying and heat‐drying in an oven at 105°C for 24 h. Ash was determined by incineration at 550°C, according to the method of the Association of Official Analytical Chemists (Association of Official Analytical Chemists, 1999). Nitrogen was determined using the rapid N cube analyzer (Elementar, Hanau, Germany), and the total content of crude proteins was calculated by multiplying carcass nitrogen by 6.25 (Association of Official Analytical Chemists, 1999; Sarr et al. 2011). All the analytical results were then expressed on a dry matter basis.

### Renal morphometric analysis

Renal structure was assessed on day 28 at the end of the neonatal period. The kidneys of each animal were rapidly harvested and weighed. The renal histology and corresponding parameters were analyzed by one investigator without prior knowledge of the group to which the piglets belonged. Half of a right kidney was fixed in 4% buffered formaldehyde. The kidneys were then dehydrated through graded alcohols and embedded in paraffin. Transverse sections through the central portion of each kidney and 4‐*μ*m‐thick sections stained with hematoxylin and eosin were obtained for light microscopic examination. In each single section of kidney, all glomeruli (i.e., superficial and juxtamedullary), sectioned through the hilum were counted and assessed for glomerular volume (GV) and glomerular fibrosis (GF) using image‐analyzing software (SAMBA 2005 Alcatel; TITN Answare, Rennes, France). The glomerular density was determined for each kidney by counting the number of glomeruli from both outer and inner cortex in five slides. The mean glomerular density was therefore determined for each kidney (Bassan et al. 2000). In each specimen, more than 100 glomerular cross‐sections not crossing the outline of the examined field were analyzed for each group, without extensive structure alterations. The profile of a glomerulus was captured and the perimeter of Bowman's capsule was traced using a tablet cursor to determine GV. Cross‐sectional tuft area (G_A_) was calculated for each glomerulus with a visible vascular pole using image‐analyzing software (SAMBA 2005 Alcatel; TITN Answare, Rennes, France). GV was then calculated, assuming the glomerulus to be spherical by applying the following mathematical equation as GV = *β*/k.(G_A_)3/2, where *β* is the shape coefficient for a sphere (=1.38) and k is the size distribution coefficient (= 1.1). Glomerular fibrosis was evaluated using Sirius red coloration to visualize fibrillar collagen (Boubred et al. 2009). The measurement of Sirius red‐stained area as the percentage of total glomerular surface area was thus evaluated. A quantitative analysis was performed by a single examiner using the same colorimetric and light thresholds (NCSS 2004 software, Kaysville, UT). Color threshold was then applied to identify the red‐stained structure. The results were reported as the mean ratio of Sirius red‐stained areas to total glomerular capillary.

### Statistical analysis

Data are presented as means ± SEM. One‐way ANOVA with a Student–Newman–Keuls comparison post hoc test analysis (Statview version 5.0 Software; Abacus Concepts, Berkeley, CA) was used to analyze differences between groups. The interaction between diet and gender was first evaluated and this did not show a gender effect; also data are presented for the overall population. Statistical significance was defined as *P *<* *0.05.

## Results

### Birth weight and neonatal growth

Birth weight, body weight growth, and tissue characteristics are shown in the Table 1. Birth weight of IUGR piglets exposed postnatally to normal‐protein (NP) formula and high‐protein (HP) formula did not differ. However, at the end of the neonatal period, HP piglets were significantly heavier, with body weight 18% higher than NP piglets (*P* < 0.01). HP piglets also showed a 22% higher protein content (*P* < 0.01) in the carcass analysis and lower PRAT mass (*P* < 0.05) than NP piglets. The PRAT relative to body weight was 40% lower.

**Table 1:** Neonatal growth and body composition features in 28‐day‐old piglets fed HP and NP formula

(Mean ± SEM)	HP	NP	*P* value
Birth weight (g),	0.94 ± 0.08	0.90 ± 0.07	0.46
Daily weight gain (g/day)	179 ± 17	146 ± 11	**0.003**
*Day 28*			
BW day 28 (kg)	5.96 ± 0.4	5 ± 0.3	**0.004**
Right carcass weight (g)	2046 ± 191	1635 ± 171	**0.01**
Protein content %	63 ± 3	49 ± 2	**0.008**
PRAT (g)	13.4 ± 1.6	18.7 ± 2.4	**0.01**
Relative PRAT weight (g/kg BW)	2.3 ± 0.4	3.8 ± 0.7	**0.01**
ST muscle, g	22.8 ± 2.8	16.8 ± 2.7	**0.01**
Relative ST muscle weight (g/kg BW)	3.8 ± 0.3	3.3 ± 0.4	0.14
Kidney weight (g)	34.2 ± 3.9	31.7 ± 3.1	0.35
Relative kidney weight (g/kg BW)	5.7 ± 0.4	6.2 ± 0.3	0.12

Bold values indicate significant *P* values

PRAT, peri‐renal adipose tissue; ST muscle, semi‐tendinus muscle

### Renal function and structure

**Figure 1:**
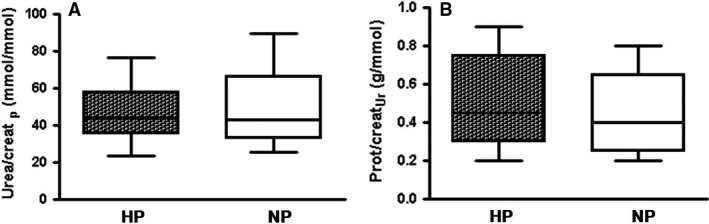
HP feeding of IUGR piglets did not affect renal workload Plasma urea/creatinine ratio (A) a surrogate of renal workload and urinary protein/creatinine ratio (B) a surrogate of SNGHF in 28‐day‐old piglets fed HP (dark plots, *n* = 10) or NP (open plots, *n* = 9) formula (median, min‐max), *P* = ns.

Parameters of the renal function are shown in the Table 2 and the Figure 1. HP intake had no significant effects on renal functions. Markers of glomerular filtration rate (GFR) were unchanged as was proteinuria (Fig. 1B). Plasma urea/creatinine ratio, which is a surrogate of renal workload attributed to high protein intake, was also unchanged in HP piglets (Fig. 1 A).

**Table 2:** Parameters of renal functions in 28‐day‐old piglets fed HP or NP formula

(Mean ± SEM)	HP	NP	*P* value
Plasma creatinine (*μ*mol/L)	78 ± 16	83 ± 21	0.68
Plasma urea (mmol/L)	3.4 ± 0.4	3.2 ± 0.7	0.30
Plasma urea/creatinine (mmol/mmol)	46 ± 12	49 ± 17	0.73
Protidemia (g/L)	51 ± 5	52 ± 5	0.89
Plasma sodium (mmol/L)	135 ± 3	136 ± 4	0.56

**Figure 2:**
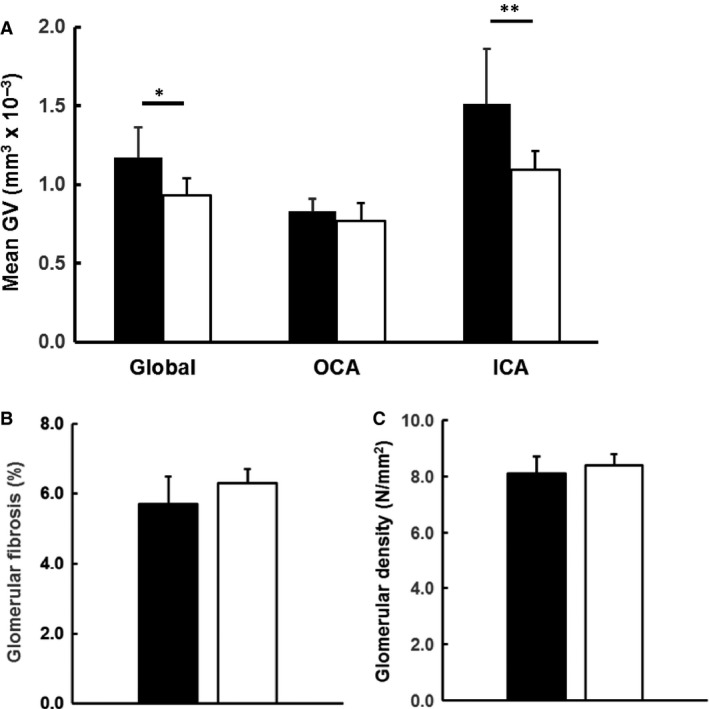
HP feeding of IUGR piglets induced mild glomerular hypertrophy restricted to nephrons of the inner cortical area. Mean glomerular volume (MeanGV) (A), glomerular fibrosis (B), and glomerular density (C) in HP (black bars, *n* = 9) and NP (open bars, *n* = 9) 28‐day‐old piglets. Inner cortical area: nephrons from the inner cortical area; outer cortical area: nephrons from the outer cortical area, *P =* *0.02; **P =* *0.03. Glomerular fibrosis and glomerular density did not differ between both groups.

Regarding the renal structure, the kidney weight of HP piglets did not differ with NP ones although they displayed a rapid neonatal growth; the relative kidney to body weight ratio was unchanged in HP piglets (Table 1). Glomerular density was not different between both groups as well (Fig. 2C). Mean glomerular volume (MGV) was elevated in HP piglets (+20% vs. NP piglets, *P* = 0.03) and restricted to inner cortical area (ICA) nephrons (Fig. 2A). MGV was higher in the nephrons of ICA in both experimental groups than in those of outer cortical area (OCA) (*P* < 0.05). Glomerular fibrosis was unaffected by HP intake (Fig. 2B).

## Discussion

This study provides further information on the renal effects of HP intake in IUGR offspring. To the best of our knowledge, this is the first study that specifically investigated such effects in spontaneous IUGR piglets which is a suitable model for human IUGR infants. We showed that feeding spontaneous IUGR piglets with a HP formula enhanced neonatal growth and tissue protein deposition without significantly affecting the renal structure. We focused on IUGR offspring since IUGR infants are more concerned with HP intake than NBW infants; other studies including NBW offspring are needed to delineate the role of nutrition and nephron deficit. However, it can be speculated that enhanced tissue protein deposition in response to HP intake limited single nephron glomerular hyperfiltration (SNGHF) in IUGR piglets.

HP feeding enhanced neonatal growth in spontaneous IUGR piglets. This growth effect was likely due to enhanced tissue protein accretion as illustrated by a higher semi‐tendinus muscle mass, lower peri‐renal adipose tissue (PRAT) deposition, and higher carcass protein content in HP piglets than in NP offspring as previously described (Sarr et al. 2011). IUGR piglets vigorously responded to HP intake (Davis et al. 2008; Jamin et al. 2010). In a similar study design, HP feeding of IUGR piglets has been shown to induce metabolic and electrolyte disturbances mimicking refeeding syndrome, a surrogate of active anabolic response to HP intake (Jamin et al. 2010).

Enhanced growth and protein tissue deposition were attributed to the higher protein intake. We used such formula to closely mimic the enriched formula used routinely for low birth weight infants with about 40–50% higher protein contents than standard formula. HP formula provided a moderate 6% increase in calories (120 vs. 113 kcal/100 mL) but 50% more proteins than NP formula. During the experiment, the individual daily milk quantity offered to the piglets was adjusted to provide the same calories intake which finally resulted in a significant high protein intake.

The main finding is that HP feeding IUGR piglets did not induce significant adverse renal changes. Indeed significant renal hypertrophy and renal overweight were expected to occur in such a situation. Few studies have investigated the effects of neonatal HP intake on the renal structure but none in piglets. In comparison with NP formula, we previously showed in IUGR rat offspring that neonatal HP formula increased kidney weight by 20% and induced renal hypertrophy with enlarged glomeruli and tubules in pups at the end of the neonatal period (Delamaire et al. 2012). In this IUGR rat experiment, glomerular hypertrophy was dramatically elevated by about 50% and concerned all nephrons located in the inner and outer cortical areas. Such an extensive glomerular hypertrophy indicates a dramatic SNGHF which involved renal solute workload, tubular amino‐acid overload responsible for passive vasodilatation of the afferent glomerular arterioles and renin angiotensin system up‐regulation (Brenner et al. 1982; King and Levey 1993; Schrijvers et al. 2002; Slomowitz et al. 2004). SNGHF is associated with renal hypertrophy and renal overweight. These effects are, moreover, amplified when associated with a nephron number deficit. (Brenner et al. 1982; Boubred et al. 2009; Myrie et al. 2011). Interestingly, both IUGR models, in the rat after maternal protein diet restriction or in spontaneous IUGR piglets, induce nephron deficit of about 30% which make them highly susceptible to HP intake (Boubred et al. 2009; Myrie et al. 2011). In contrast with HP‐fed IUGR rat, HP‐fed IUGR piglets only had a 20% increase in MGV restricted to nephrons from the inner cortical area without other changes in renal structure and renal functions. This change depicts a moderate SNGHF since structure of outer nephron was unaltered. Indeed, postnatal maturation is associated with a progressive increase in glomerular filtration rate and glomerular and tubular enlargement of nephrons from the inner to the outer cortical area. Moreover, differences in MGV are unlikely attributable to nephron number variation since birth weight, kidney weight, and glomerular density were not different between NP and HP piglets. All of these findings support a mild SNGHF in HP IUGR piglets.

Enhanced tissue protein deposition may have mitigated the adverse renal consequences of HP intake in IUGR piglets. One would expect severe adverse renal disease in HP IUGR piglets especially as they had a rapid neonatal growth. We speculate that appropriate protein utilization for tissue growth limited SNGHF. Despite a HP intake, plasma urea concentrations and plasma urea/creatinine ratio, a surrogate of renal workload, did not increase. Renal overgrowth was not found as well. In our previous work, HP feeding in IUGR rat offspring failed to improve net protein gain but induced kidney overweight and renal injury as early as the neonatal period (King and Levey 1993; Sarr et al. 2011). Even though caution is required when comparing both IUGR model in which metabolic responses to HP intake may be differentially altered after maternal protein diet restriction, all of these findings suggest that the body capacity to metabolize dietary proteins may mediate the renal effects of HP intake (Sarr et al. 2011; Delamaire et al. 2012). More accurate assessment of protein metabolism will confirm our hypothesis.

In this study, we chose to investigate the effects of a neonatal HP diet in IUGR offspring. This study followed on our previous works in which we demonstrated that neonatally overfed and HP formula‐fed IUGR rat offspring were more susceptible to renal diseases than NBW offspring (Boubred et al. 2009, 2016). Our study was not designed to evaluate the effect of a HP diet in NBW piglets. Another study, using a similar model, showed unchanged kidney weight in NBW piglets fed with milk with 50% increase in protein content (Han et al. 2013); renal functions and structure were not investigated unfortunately. In this study, we did not find differences in GFR between both groups. This is due to low accuracy of plasma creatinine concentrations to evaluate GFR. This marker is also inappropriate to evaluate single nephron glomerular filtration rate (SNGFR); but is commonly used in clinical practice. Anyway, if differences existed between both nutrition groups they would be small and SNGFR would be less likely to increase dramatically since renal structure and urinary protein concentrations, a surrogate of SNGHF, were mildly altered or unchanged in HP piglets. Finally, we focused on the neonatal period but it is not excluded that these mild functional and structural changes can evolve toward significant complications later at adulthood as observed in our previous work. Additional investigations should be conducted to assess long‐term vascular and renal consequences in adult offspring.

### Clinical implication

Caution is required when extrapolating our findings to human IUGR since current findings were observed in a specific experimental model; but evidence argues for the potential relevance for human IUGR. In humans, placental insufficiency is the leading cause of IUGR. In the current experimental model, spontaneous IUGR piglets result from placental insufficiency with reduced transplacental amino acid transfer and fetal glucocorticoids overexposure as observed in humans (Bauer et al. 2003). Metabolic responses to high protein intake may differ between piglets and newborn infants with higher protein accretion rate in piglets (Han et al. 2013). But as found in IUGR piglets, some IUGR infants can display accelerated growth and metabolic disturbances mimicking refeeding syndrome in response to HP intake (Jamin et al. 2010; Boubred et al. 2015 Nov). Regarding the kidney, two recent studies reported renal hypertrophy (+10% to +25% increase in kidney volume) in 3‐ and 6‐mo‐old NBW infants exposed to HP formulas from birth onward (Schmidt et al. 2004; Escribano et al. 2011). In one of them, renal hypertrophy was associated with increased renal workload marker (almost twofold increase in blood urea/creatinine ratio) indicating that a part of dietary proteins intake was not used for tissue growth and oxidized to urea (Escribano et al. 2011). However, no study has appropriately explored the renal consequences of HP intake in IUGR infants.

## Conclusion

In conclusion, in spontaneous IUGR piglets, HP intake enhanced neonatal growth and tissue protein deposition without severely affecting renal structure and functions in the neonatal period. Long‐term renal consequences remain to be investigated. The underlying mechanism of such unexpected mild adverse renal effects is unknown. It can be speculated that enhanced tissue protein deposition in response to HP intake limited single nephron glomerular hyperfiltration in IUGR piglets. This hypothesis has to be tempered in the absence of NBW offspring exposed or not to HP formula. Development of early biomarkers of SNGHF and protein metabolism may help the clinician to guide nutritional support in LBW and IUGR infants.

## Conflict of Interest

Authors have no conflict of interest.
